# A novel method to treat recurrent facial pain: a case report

**DOI:** 10.1186/s13256-021-02888-1

**Published:** 2021-07-01

**Authors:** Jintakorn Kuvatanasuchati, Karoon Leowsrisook

**Affiliations:** 1 Department of Microbiology, Faculty of Dentistry, Chulalongkorn University, Bangkok, Thailand; 2 Walailak University International College of Dentistry, Walailak University, Nakhon Si Thammarat, Thailand

**Keywords:** Occlusal equilibration appliance (OEA), Pain Posselt’s finding, Recurrent chronic facial pain

## Abstract

**Background:**

Chronic facial pain is a serious condition affecting millions of people worldwide. The reasons for chronic facial pain vary, and currently, the methods of treating chronic facial pain are expensive, invasive, and, based on current findings, ineffective. The purpose of this study is to develop and test an effective, cost-friendly method to treat patients with chronic facial pain. This study will examine the effectiveness of a novel treatment of a patient suffering from trigeminal neuralgia.

**Case presentation:**

A 70-year-old Thai female visited the advanced general dentistry clinic at the Faculty of Dentistry, Mahidol University, Bangkok, Thailand. She was suffering from facial pain on her left side and was diagnosed by a physician as having trigeminal neuralgia. She experienced a sharp shooting pain that was triggered by facial movements such as chewing, speaking, or brushing teeth, and touching certain areas of her face. Bouts of pain lasted from a few seconds to several minutes, and episodes of several attacks lasted days, weeks, months, or longer prior to her visit to the advanced general dentistry clinic at Mahidol University. Physician designed an occlusal equilibration appliance for treating the patient by inserting the appliance in the mouth for dental occlusal equilibration (deprogram). The patient used this appliance by placing it in the mouth continuously (day and night) and removed it only when eating. After using the appliance for 2 weeks, the patient appeared to feel and look better prior to taking medication and was able to eat normally. The patient was pain free after treatment for a duration of 9 months. However, after 9 months, the pain reoccurred and manifested itself.

**Conclusion:**

This novel treatment of recurrent facial pain showed an improvement of the patient’s chronic facial pain and serves as evidence to being a novel method for treating those suffering from trigeminal neuralgia.

**Supplementary Information:**

The online version contains supplementary material available at https://doi.org/10.1186/s13256-021-02888-1.

## Background

Chronic idiopathic orofacial pain is a misunderstood group of conditions that may involve the entire mouth and face. Unfortunately, descriptions of disorders and treatments tend to be influenced by the background of the specialist assessing the patient. Thus, patients who seek advice from maxillofacial surgeons have symptoms described in terms of clicking, sticking, and locking of the temporomandibular joint and pain in the associated musculature. Ear, nose, and throat surgeons may retain Costen’s outdated notion that the pain is due to missing molar teeth and may refer to maxillofacial surgeons or restorative dental specialists. Despite advice from the National Institute of Health (NIH) that “there is no evidence linking occlusal abnormalities with pain,” patients’ occlusions continue to be adjusted by ill-informed practitioners, often leading to more problems for patients [1].

The NIH conference in 1996 reviewed the issues related to the management of orofacial pain, concluding that major problems hampered present diagnostic classifications and treatment. Five years later, there is no greater clarity in classification. Current diagnoses include tension headache, migraine, neck pain, temporomandibular disorder (temporomandibular joint pain dysfunction syndrome, facial arthromyalgia), and atypical facial pain. These pains seem to arise from blood vessels, muscles, and joint capsules rather than conforming to the distribution of sensory nerve branches, as in trigeminal neuralgia. Artificial distinctions in clinical presentation lead patients to different specialists providing different treatments, including dentists, neurologists, otolaryngologists, osteopaths, chiropractors, and psychiatrists, with little collaboration. There are additional important problems concerning the recognition and definition of underlying psychiatric disturbances. Emotional disturbance, when present, is often mild and of brief duration, and psychiatric classification has proved to be an inadequate measure. Chronic pain is one of the main causes of physical and psychosocial distress, absences at work, and retirement due to handicap [2, 3]. It causes intense suffering, anxiety, and incapacitation [4]. Patients often need an interdisciplinary group, and the focus of treatment should include quality of life and coping [5]. Therefore, there might be differences among the diseases, and chronic pain should be investigated not only in its physical but also in its psychological aspects. Trigeminal neuralgia (TN) is an excruciating neuropathic pain with unknown etiology, and it is considered one of the worst causes of pain-associated suffering [6]. It often causes depression and even suicide in some cases. Its treatment is usually efficient in the beginning, with carbamazepine as the drug of choice [7]. Unfortunately, more than 75% of patients need neurosurgery to control their pain during the first 5 years [8]. The long history of pain and return of crises are important factors that indicate the need of support for these patients. Even after surgery, many of them have complications including numbness and masticatory abnormalities, such as chewing difficulties, weakness at the jaw, mouth-opening limitations [9], and reoccurrence of pain. Temporomandibular disorder (TMD) is a general term for musculoskeletal pain of the masticatory system with multiple etiology. It is one of the most common diagnoses of chronic orofacial pain, associated with psychosocial, behavioral, cognitive, and emotional factors [10], and patients often have depression and/or anxiety. Beyond physical abnormalities at the muscles and at the teeth and joints, it is also associated with emotional stress [11], and psychological assistance is necessary for most of the patients [12]. Different orofacial pains may cause variable levels of anxiety and depression, and various coping strategies, daily limitations, or perception of the disease.

## Case presentation

### History

A 70-year-old Thai female visited the advanced general dentistry clinic at the Faculty of Dentistry, Mahidol University, Bangkok, Thailand. She was suffering from facial pain on her left side and was diagnosed by a dentist as having trigeminal neuralgia (TN). This diagnosis was made shortly after pain onset, solely based on its character and without any pathologic findings from a magnetic resonance imaging (MRI) scan that the patient had undergone 9 years ago. The patient experienced flank pain, and the physician prescribed MRI for the abdominal area only. She experienced a sharp shooting pain, which was triggered by facial movements, such as chewing, speaking, or brushing teeth, and touching certain areas of her face. Bouts of pain lasted from a few seconds to several minutes, and episodes of several attacks lasted days, weeks, months, or longer prior to her visit to the advanced general dentistry clinic at the dentistry clinic at Mahidol University (Additional file 1:Fig. S1).

### Examination

Physician designed an occlusal equilibration appliance (OEA) (Additional file 1: Fig. S1) for treating the patient by inserting the appliance in the mouth for dental occlusal equilibration (deprogram). The patient used this appliance by placing it in the mouth continuously (day and night) and removed it only when eating. After using the appliance for 1 week, the facial pain had not yet disappeared. At the second week, the physician prescribed Arcoxia (etoricoxib) to inhibit cyclooxygenase-2 (Cox-2). The patient was prescribed 90 mg, 10 tablets, 1 tablet/day after meal for 10 days. After completing the full dosage prescribed, the patient appeared to feel and look better prior to taking medication and was able to eat normally. Physician did not appoint oral rehabilitation owing to lack of team resources and setup. In addition, patient did not follow the physician’s instructions on preventing oral pain by adjusting eating habits. This included abstaining from foods that are hard, crunchy, chewy, or sticky. The physician recommended instead to focus on eating creamy foods or foods that did not require a lot of mouth movement to reduce all actions of the muscle of mastication during the period before the start of oral rehabilitation.

## Results

The patient was pain free after treatment for a duration of 9 months. However, after 9 months, the pain reoccurred and manifested itself. During the period of relief, no appointment was set for oral rehabilitation owing to lack of team resources and setup. In addition, the patient did not follow the physician’s instructions on preventing oral pain by adjusting eating habits. This included abstaining from foods that are hard, crunchy, chewy, or sticky. The physician recommended, instead, to focus on eating creamy foods or foods that did not require a lot of mouth movement to reduce all actions of the muscle of mastication during the period before the start of oral rehabilitation.

**Fig. 1:**
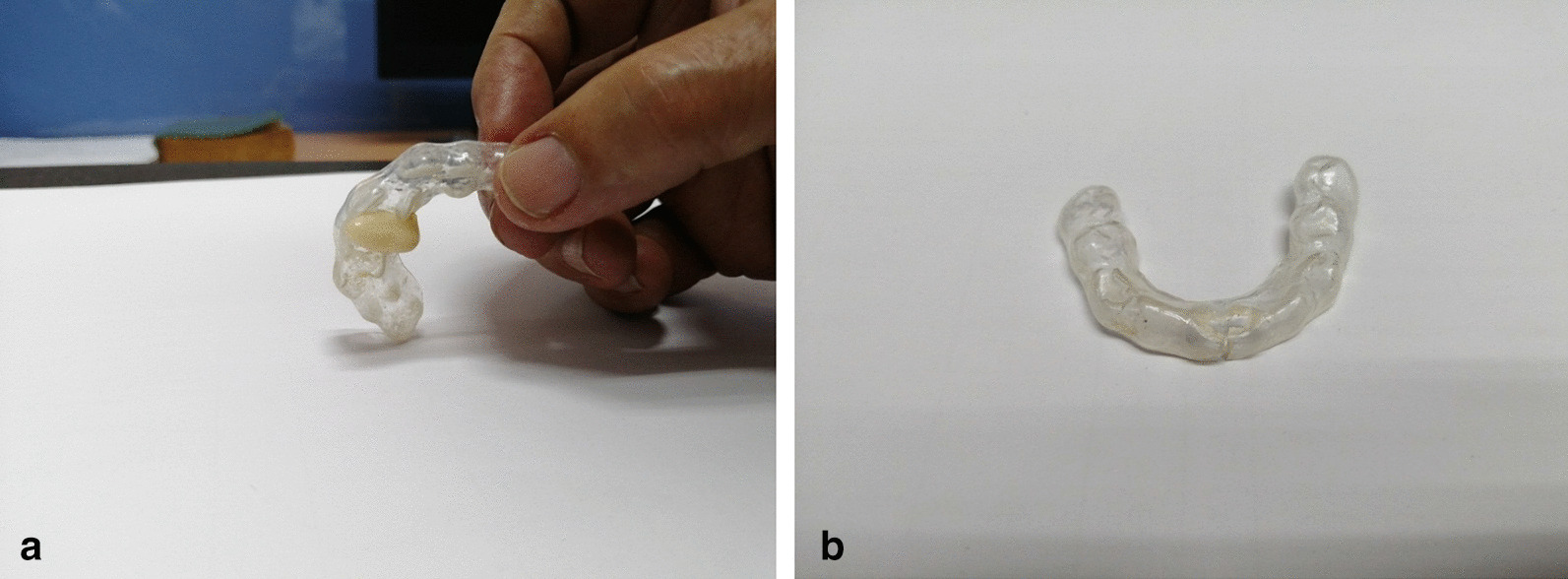
**a** Occlusal equilibration appliance abrasion (upper occlusal equilibration appliance). **b** OEA abrasion (lower occlusal equilibration appliance)

**Fig. 2:**
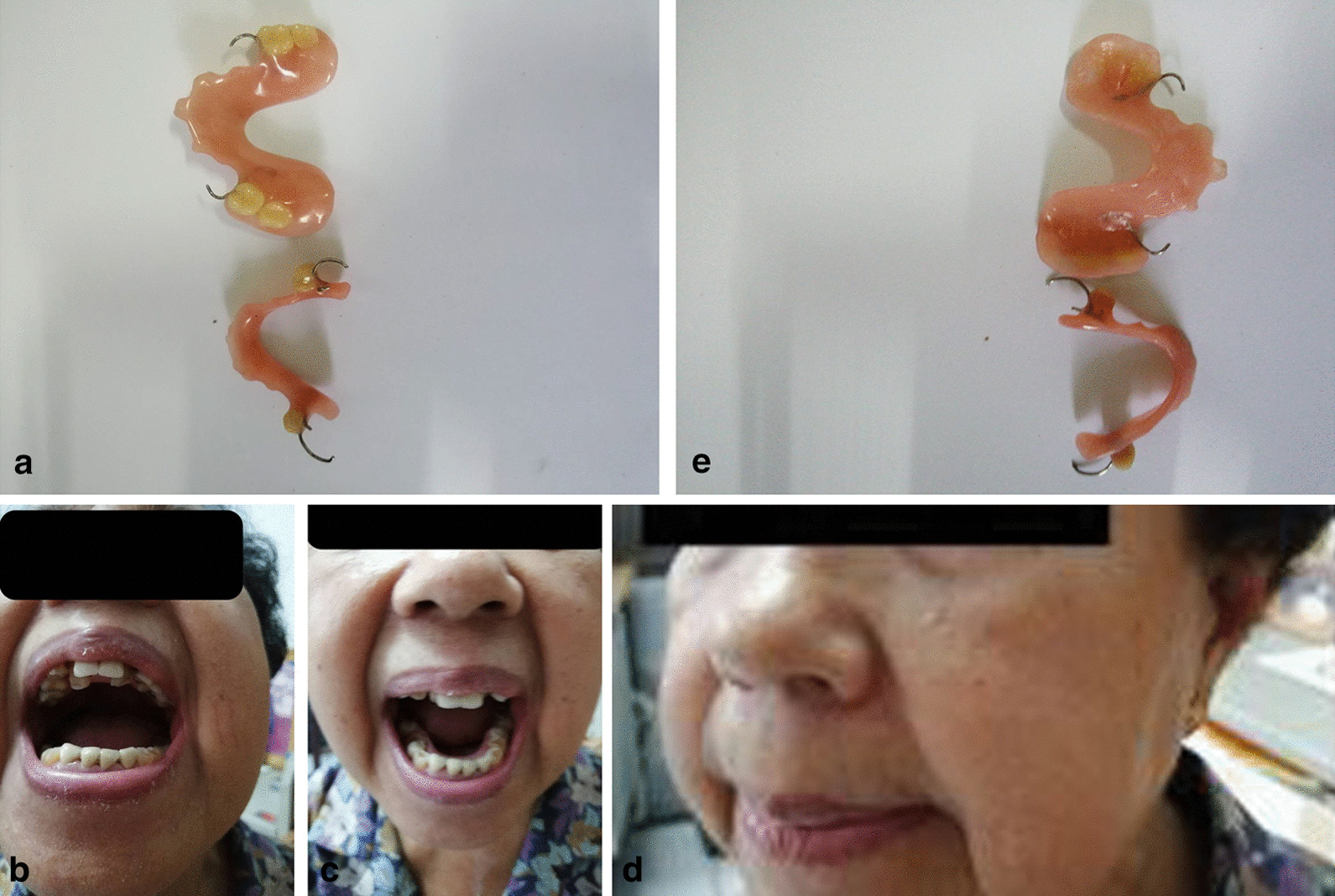
**a** Outside of upper partial denture (upper figure) and outside of lower partial denture (lower figure). **b** Patient shown with upper partial denture. **c** Patient shown with lower partial denture. **d** Front view of patient with upper and lower partial denture. **e** Inside of upper partial denture (upper figure) and inside of lower partial denture (lower figure)

The patient had pain onset on 24 August 2019. Physician prescribed Arcoxia (etoricoxib), 90 mg, 10 tablets, 1 tablet/day after meals for 10 days and recommended focusing on consuming foods that would not require much mouth movement. Currently, the patient is following the physician’s instructions on preventing oral pain by adjusting eating habits. In addition, abrasion was found in the OEA that the patient used. (Fig. 1a, b) A new OEA for the patient was made after the patient was pain free by designing a partial denture that has OEA-like qualities. (Fig. 2a–e)

Before pain onset on 24 August 2019, the patient experienced mild facial pain on her left side on 31 July 2019. The pain gradually intensified on 24 August 2019. The physician insisted on adjusting eating habits. The physician prescribed Arcoxia (etoricoxib), 90 mg, 10 tablets, 1 tablet/day after meals for 10 days. She also had acupuncture and afterwards experienced intense pain in the morning of 26 August 2019. The pain was so intense that she was unable to sleep normally, only by sitting upwards. On 28 August 2019, the physician prescribed Arcoxia (etoricoxib), 90 mg, 10 tablets, 1 tablet/day after meals for 10 days again. However, the pain did not subside and was very intense each evening. The pain was intermittent, disappearing for a few minutes and recurring continuously. On 2–4 September 2019, the patient was still experiencing pain in the evening. But on 5 September 2019, the pain started to occur during the day while brushing teeth. The patient was unable to touch her face and could not eat food, in contrast to the previous day where she was eating normally. On 6 September 2019, physician prescribed Arcoxia (etoricoxib), 90 mg, 10 tablets, 1 tablet/day after meals for 10 days again. Pain improved on 15 September 2019, but she still had pain on the left side when cleaning her face. On 18 and 19 September 2019, she had pain around the mouth in the morning but later improved in the evening. However, she was not able to consume solid foods, only liquids by sipping. The patient’s weight decreased by 10 kg during that period. On 22 and 23 November 2019, she was admitted to a private hospital, and the physician gave saline solution and recommended MRI of the brain and cranial nerve and MRA of the brain and neck. MRI found:

1. Possible vascular loop to the left CN V (looping and causing mass effect but not definite).
2. No mass or brain stem lesion.
3. Nonspecific mucosal thickening in the left maxillary sinus.
4. MRA study.

*Abrupt stenosis at V4 segment of the right VA; vertebral dissection cannot be excluded. *Diffuse mild irregularity of the intracranial vessels.

*No aneurysm or vascular malformation.

Physician also prescribed:

- Amitriptyline (Polytamol) 10 mg tab 50 tabs 1 tab before bed.
- Sermion (nicergoline) 10 mg tablets, 180 tablets, 2 tablets after meal in the morning and evening.
- Trileptal (oxacarbazepine) 300 mg tablets, 100 tablets, 1 tablet after meals, morning and evening.

The patient was also prescribed Boost Optimum and Ensure. On 22–25 November 2019, pain around the mouth improved; however, she was still unable to eat solid foods, and she explained to the physician that she had toothache at the left second premolar. The physician said that it may be pain referred from retained root (RR). After extraction of RR of left first and second molar and RR of right second molar she was able to eat food normally.

The physician performed oral rehabilitation by keeping vertical dimension normal by making a new OEA for the patient by designing a partial denture that had OEA-like qualities. (Fig. 2a–e). The OEA appliance was designed on the basis of Posselt’s finding [13] (Additional file 1: Fig. S1) of nerve entrapment in the lateral pterygoid muscle [14, 15] (Additional file 1: Fig. S2), centric relation [16]( Additional file 1: Fig. S3), and vertical dimension [13].

Posselt’s findings can briefly be summarized as follows:[5]

The movement area of the mandible in the sagittal and horizontal planes is characteristic of the individual but varies between people. However, the border movement paths are reproducible in the same individual. It is suggested that the capsules and capsular ligaments of the temporomandibular joint limit the border movement of the mandible. It is possible for the mandible to perform a posterior hinge-opening and hinge-closing movement. If the opening exceeded 25.8 ± 2.2 mm. (Additional file 1: Fig. S4), a forward-downward shift of the condyles occurred. The habitual path of closure follows a course anterior to the posterior path. Positions obtained in habitual closing are posteriorly farther when the head and/or the trunk are reclined.

The rest position (Additional file 1: Fig. S5) did not appear to be an extreme posterior mandibular position, and the intercuspal position was even more rarely so. Shifting of the mandible from rest to intercuspal position generally involves bodily movement of the mandible. Differences in the movement limitations of the mandible vary with the difference in degree of posterior bite-opening. This work is recommended to orthodontists, prostheticians, and practicing dentists in general who are interested in preserving and restoring the human masticatory apparatus. Normally, in maximum contact, condyles move downward and forward from centric relation position (slightly in centric) 0.2–2 mm (Additional file 1: Fig. S5). The maximum intercuspation causes the movement of condyle from centric relation. With this appliance, the anterior raise bite will disclude for posterior teeth. Therefore, the condyle moved freely back to centric relation by the release of lateral pterygoid muscle to the completely inactive state (Additional file 1: Fig.S1).

## Discussion

It is believed that some cases of temporomandibular joint syndrome or atypical facial pain may be due to entrapment neuropathies in the infratemporal fossa [17]. The posterior trunk of the mandibular division of the trigeminal nerve normally descends deep into the lateral pterygoid muscle. The study of nerve entrapment in the lateral pterygoid muscle by “Barry A (Loughner BA)” found that 3 of 52 dissections of the three main branches of the posterior trunk (lingual, inferior alveolar, and auriculotemporal nerves) were observed to pass through the medial fibers of the lower belly of the lateral pterygoid muscle. The mylohyoid and anterior deep temporal nerves were also observed to pass through the lateral pterygoid muscle in other specimens. These nerve entrapments in the infratemporal fossa provide new information concerning the anatomic and clinical relationships between the mandibular nerve and the lateral pterygoid muscle. These findings support the hypothesis that a spastic condition of the lateral pterygoid muscle may be causally related to compression of an entrapped nerve that leads to numbness, pain, or both in the respective areas of nerve distribution [17].

Prosthodontic treatment planning may require articulation dental casts following registration of jaw transfer records. A reproducible and stable jaw relationship is a desirable reference point for cast analysis, case planning, and subsequent treatment. The anterosuperior condyle position with an appropriately aligned interarticular disc approximating the articular eminence has been an acceptable reference position for the jaw at a clinically acceptable vertical dimension [13]. It has been suggested that condylar position may be achieved by the coordinated activity of the lateral pterygoid muscles during jaw closure and that deflective occlusal interferences may influence the recording of a reproducible jaw position [16, 18].

The use of an anterior jig (AJ) [19], Roth’s power “centric relation” registration [20], or a leaf gauge (LG) [21] are techniques that have been proposed for jaw transfer records to achieve a superior positioning of the condyles. These techniques used an anterior stop to disclude posterior teeth and eliminate possible tooth contact interferences. However, there is no consensus on which technique allows the recording of this patient-specific condylar position. Biting on an AJ and an LG effectively removes potential occlusal interferences and separates the posterior teeth. Current understandings of jaw muscle physiology have shown that incisal biting reduces the level of bite force and alters jaw-closing muscle EMG (electromyographic) activity. Jaw recording should allow a consistent position to be obtained as a condylar reference position for each patient. The use of an AJ and an LG should allow a measurable displacement of the condyle, compared with intercuspal clench. Understanding the implications of these clinical procedures and their possible effects on condylar displacement and associated jaw muscle activity should provide objective clinical data to justify the use of a recorded position as a reproducible treatment position (Additional file 1: Fig. S6). To release contraction of lateral pterygoid muscle, the physician designed a partial denture that has OEA-like qualities (Fig. 2a–e). This appliance supported the condyle to move back to the centric relation according to Posselt’s border of movement theory [13]. Currently, the patient is following the physician’s instructions on preventing oral pain by adjusting eating habits and using the denture with OEA-like qualities only at night when sleeping by placing it in the mouth. The patient prefers not to wear the denture throughout the day because of discomfort. The patient has experienced less sensitivity around the face, and she is able to eat food normally. This article will continue to follow up with the patient’s progress.

## Conclusion

We report a case of a 70-year-old Thai female who visited the advanced general dentistry clinic at the Faculty of Dentistry, Mahidol University, Bangkok, Thailand. The patient was pain free after treatment for a duration of 9 months. However, after 9 months, the pain reoccurred and manifested itself. Currently, the patient is following the physician’s instructions on preventing oral pain by adjusting eating habits, and the findings from the study show an improvement in the patient’s chronic facial pain, serving as evidence of being a new method for treating. With further studies, it may prove useful in helping the millions of people suffering from trigeminal neuralgia. This article will continue to follow up with the patient’s progress.

## Supplementary Information

**Additional file 1.** Additional figures.

## Data Availability

The data analyzed during the current study are available from the corresponding author on reasonable request.

## Abbreviations

TN: Trigeminal neuralgia
MRI: Magnetic resonance imaging
OEA: Occlusal equilibration appliance
NIH: National Institutes of Health
TMD: Temporomandibular disorder
Cox-2: Cyclooxygenase-2
MRA: Magnetic resonance angiography
CN V: Trigeminal nerve
VA: Vertebral arteries
V4: Intracranial segment of VA
RR: Retaining root
LG: Leaf gauge
AJ: Anterior Jig
EMG: Electromyographic
