# Supplementary material for: A novel method to treat recurrent facial pain: a case report
Source: J Med Case Rep. 2021 Jul 1;15:337. doi: 10.1186/s13256-021-02888-1 (PMC8247074; doi:10.1186/s13256-021-02888-1)
Supplement: Supplementary file 1 — Additional file 1. Additional figures. [file 13256_2021_2888_MOESM1_ESM.docx]

**The treatment of recurrent chronic facial pain**

Jintakorn Kuvatanasuchati^1,2^, DDS, MS, Karoon Leowsrisook^2^, DDS, MS.

^1^Department of Microbiology, Faculty of Dentistry, Chulalongkorn University, Bangkok, Thailand.

^2^Walailak University International College of Dentistry, Walailak University, Nakhon Si Thammarat, Thailand.

**Author for correspondence:** Jintakorn Kuvatanasuchati E-mail: [Jintakorn.ku@wu.ac.th](mailto:Jintakorn.ku@wu.ac.th), [Jintakorn.k@chula.ac.th](mailto:Jintakorn.k@chula.ac.th) Tel: +662-2980244 ext.1138 ; Fax:+662-2990836,


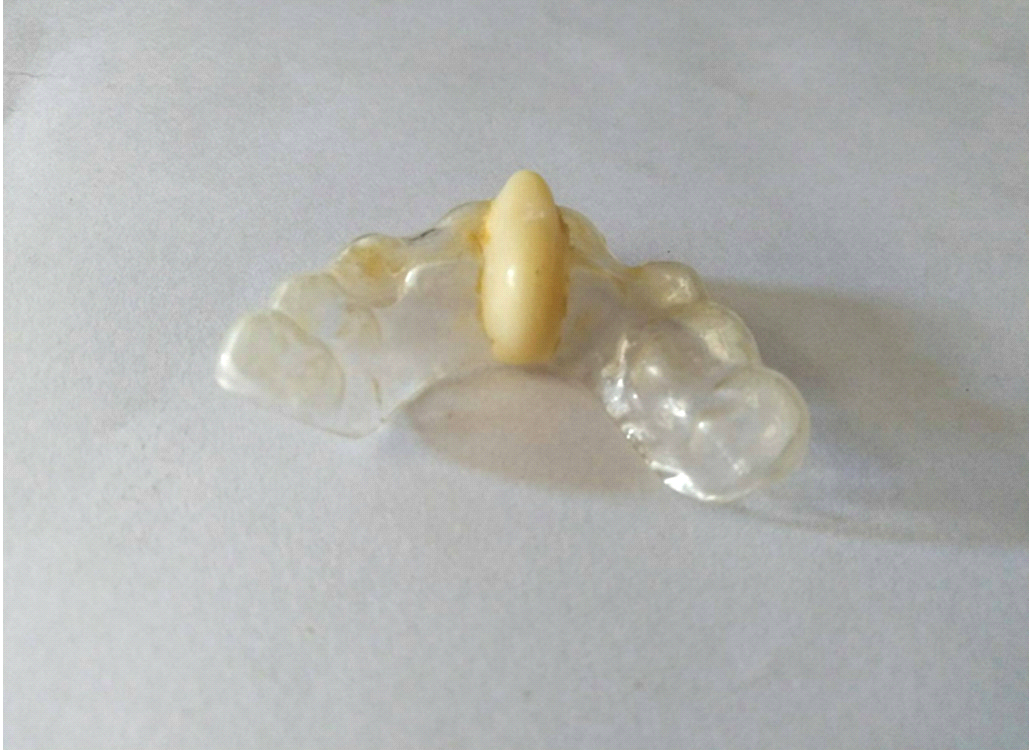


**Fig. S1** Occlusal equilibration appliance (OEA), which is base on Posselt’s finding [6], Nerve entrapment in the lateral pterygoid muscle [7], centric relation [8] and vertical dimension [6].

**Fig. S2** Nerve entrapment in lateral pterygoid muscle


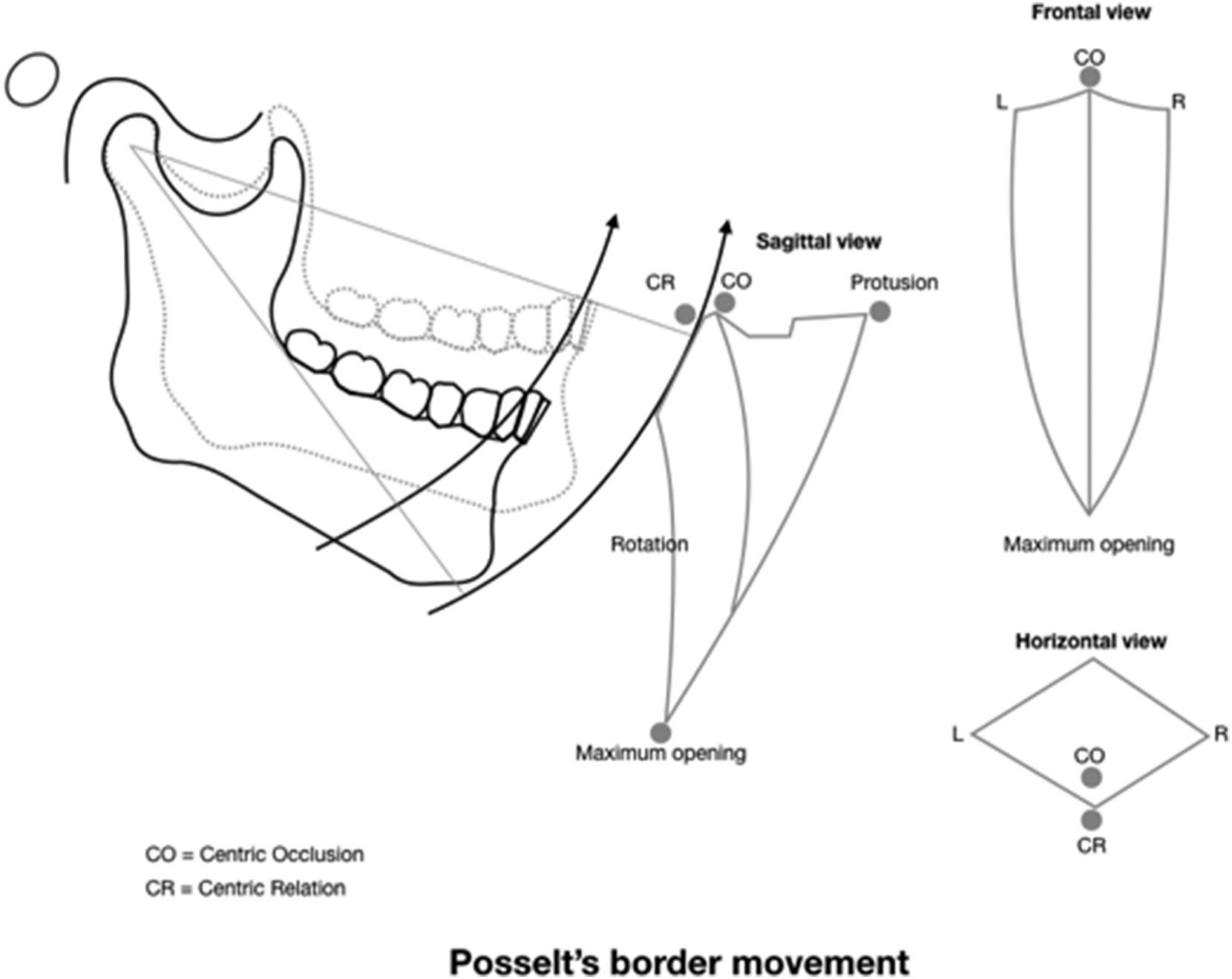


**Fig. S3** Posselt’s border movement


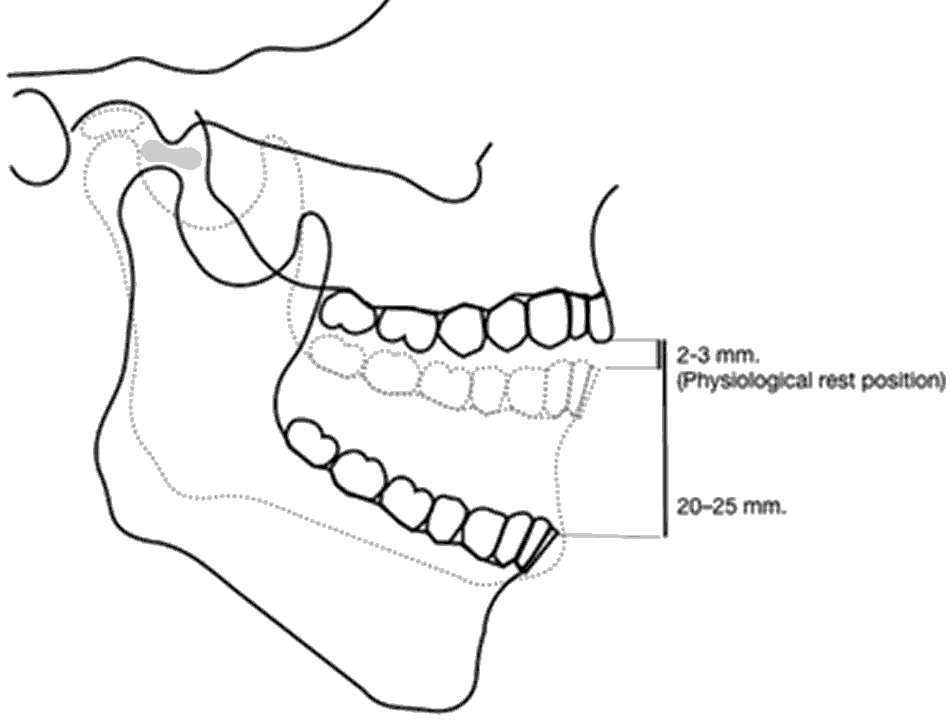


**Fig. S4** Translation or gliding movement

-Translation is the bodily movement of the head of the condyle. It takes place in the upper compartment of the TMJ between the superior surface of the articular disc as it moves with the condyle and the inferior surface of the glenoid fossa.

-If opening of the mandible continues beyond 20–25 mm then translation of the mandible occurs.


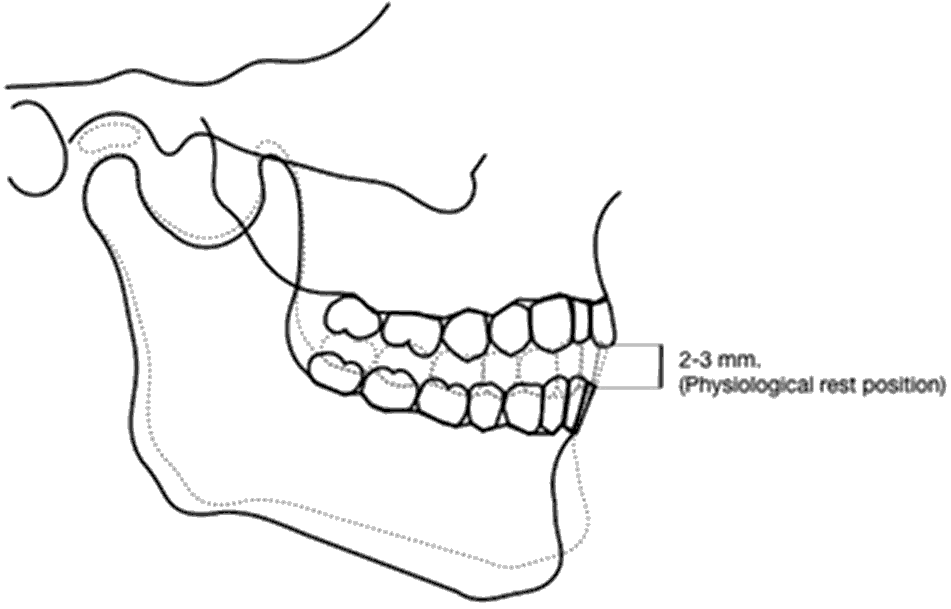


**Fig. S5** Physiologic rest position


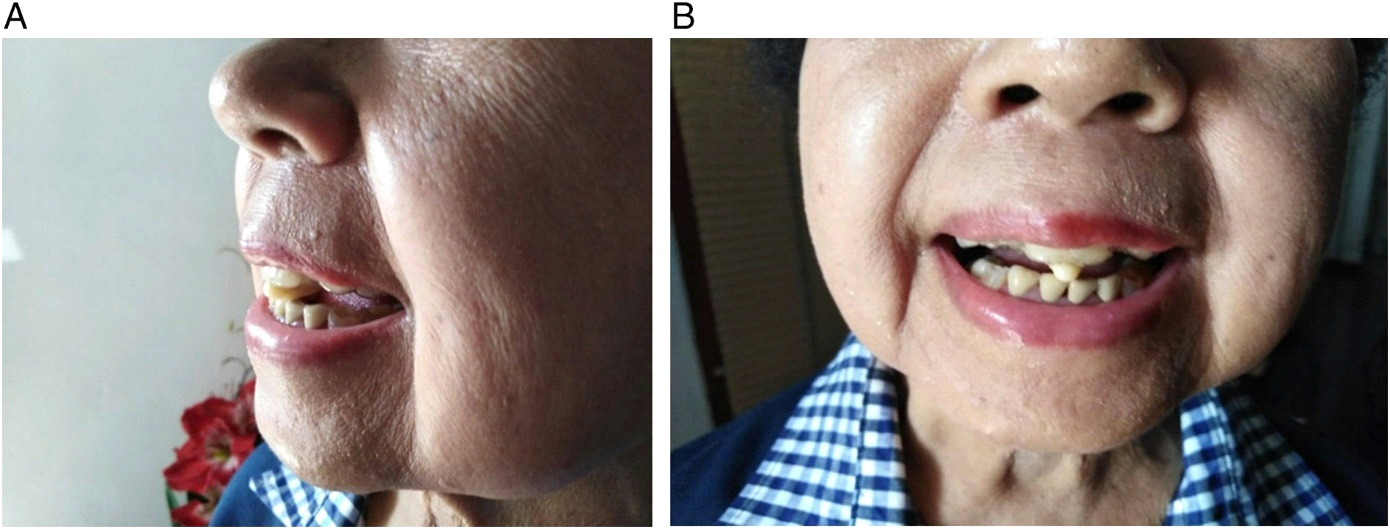


**Fig. S6** Patient’s picture, Side view (A); Front view (B)
